# A Devastatingly “Minor” Relationship Between Male Breast Cancer and Prostate Cancer

**DOI:** 10.7759/cureus.3463

**Published:** 2018-10-17

**Authors:** Sindhura Kolli, Armand Asarian, Romulo Genato, Philip Xiao

**Affiliations:** 1 Internal Medicine, The Brooklyn Hospital Center, Brooklyn, USA; 2 Surgery, The Brooklyn Hospital Center, Brooklyn, USA; 3 Pathology, The Brooklyn Hospital Center, Brooklyn, USA

**Keywords:** male breast cancer, palb2, nbn, brca1, brca2, prostate cancer

## Abstract

Certain cancers pave way for other primary cancers to emerge with genetic disturbances serving as a common denominator as demonstrated by our male patient who developed prostate cancer within three months of being diagnosed with breast cancer despite being negative for the major genetic mutations, BRCA1 and BRCA2 and having a negative family history for cancers. Here we examine overlapping major and minor contributing risk factors and the limitations of the most current screening guidelines.

## Introduction

Breast cancer in males is a relatively rare occurrence compared to in females, accounting for less than 0.003% of cancers that affect men. However, the incidence of these male breast cancer patients developing a second primary cancer is surprisingly high at an incidence of 11.5% [1], echoing our own patient’s unfortunate sequelae of developing prostate cancer within three months of breast cancer. Given the infrequency of these singular cases, there are limited and relatively naive standardized screening guidelines tailored to address the presence of minor genetic mutations such as PALB2 and NBN versus major ones such as breast cancer early onset protein 1 and 2 (BRCA1 and BRCA2) in the prevention of a second primary cancer, especially in males. This forces us to look at secondary risk factors such as age at time of diagnosis, stage of breast cancer, hormone receptors along with minor genetic mutations for partial illumination on whether these patients are at risk for developing a second cancer, such as prostate cancer.

## Case presentation

**Figure 1:**
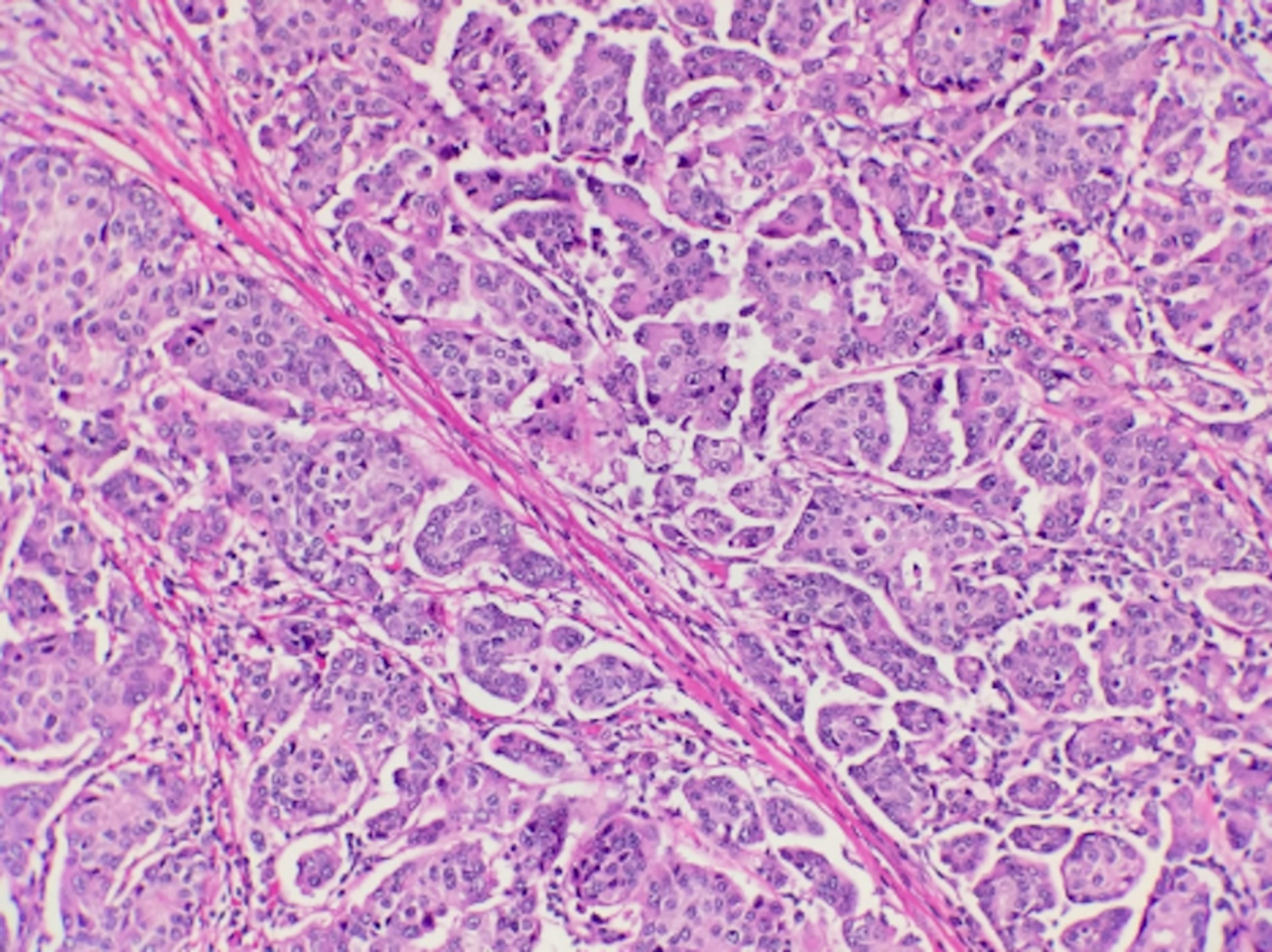
Microscopic examination of ultrasound-guided breast biopsy reveals infiltrating glandular malignant cells.

A 64-year-old African American male patient with no significant family history and recently diagnosed poorly differentiated invasive ductal carcinoma and ductal carcinoma-in-situ was referred to our institution after a mammogram showed that the breast cancer had increased in size from 2.2 to 2.6 cm and detection of new abnormal axillary lymph nodes. Ultrasound-guided core biopsies of the palpable right axillary lymph nodes showed that the primary breast cancer had metastasized to the nodes (Figure 1) and the tissue was positive for antibodies to estrogen receptors or anti-estrogen receptors (ER) and antibodies to progesterone receptors or anti-progesterone receptors (PR), but negative for antibodies to human epidermal growth factor receptor 2 (Her2/neu) receptors.

Genetic testing yielded negative BRCA1/BRCA2, however was positive for heterozygous partner and localizer of BRCA2 (PALB2) c.3027del gene and variants of nibrin (NBN) c.1354A>C and c.511A>G genes. The patient underwent a right modified radical mastectomy with appropriate follow-up.

**Figure 2:**
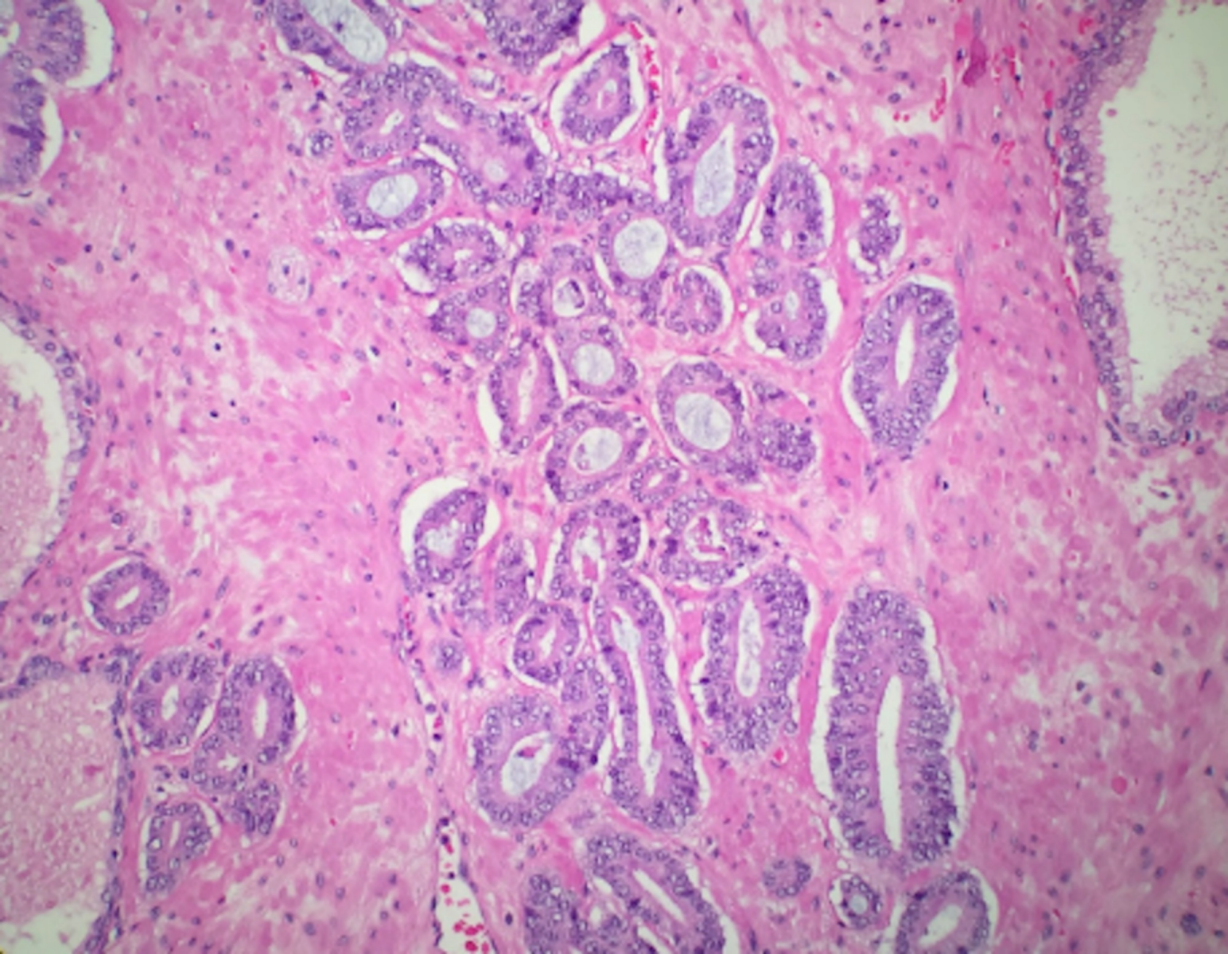
Microscopic examination of prostate biopsy reveals prostate malignant glands with Gleason grade 6 pattern.

The patient returned within three months of his mastectomy with symptoms of prostate enlargement which included difficulty urinating and retention and was subsequently found to have an elevated prostate-specific antigen (PSA) >12. The patient underwent a robotic laparoscopic radical prostatectomy with bilateral pelvic node dissection in which biopsies of the prostate and pelvic nodes demonstrated prostatic adenocarcinoma Gleason grade seven with capsular involvement (Figure 2). No vascular involvement was detected and surrounding pelvic nodes were negative for carcinoma. Currently, the patient is undergoing chemotherapy with adriamycin which inhibits topoisomerase and cyclophosphamide, an alkylating agent, to be followed by paclitaxol which inhibits microtubule function.

## Discussion

Studies previously conducted with an objective to uncover an association between male breast cancer and prostate cancer showed a significant relationship between prostate cancer and male breast cancer regardless of the presence of major gene mutations [2]. The risk of developing prostate cancer had a higher incidence within 12 months of the initial breast cancer diagnosis and more prevalent in younger men aged 65–74 years at the time of breast cancer diagnosis versus in men older than 75. Prostate cancer occurrence was markedly higher in those with stage 1 breast cancer indicating invasive breast cancer confined to a single breast and hormone receptor-positive breast cancer, which includes anti-estrogen receptors (ER), anti-progesterone receptors (PR), and antibodies to human epidermal growth factor receptor 2 (Her2/neu) [3]. Our patient was positive for multiple risk factors being 64-year-old at the time of diagnosis of breast cancer, developed prostate cancer within three months of initial breast cancer diagnosis, and was ER and PR positive. However, none of these risk factors indicated additional screenings to prevent prostate cancer, but were noted in hindsight.

The presence of genetic mutations would have been a reason for increased and earlier surveillance screening for prostate screening. Major genetic mutations implicated in the development of breast cancer, such as BRCA1 and BRCA2, increase the incidence of a second primary cancer. This reinforces the current 2018 National Comprehensive Cancer Network (NCCN) guidelines for breast and ovarian cancer which state that prostate screening for BRCA2 male carriers should start at the age of 45 [4], but little is mentioned in the absence of a BRCA status. BRCA2 carriers have an 8.6-fold increase in developing prostate cancer if under the age of 65 and they have a 20% lifetime risk through loss of its tumor suppressor function. BRCA1 mutations, while less common in male breast cancer cases versus female breast cancer cases, increases the risk of prostate cancer by 3.5-fold in men under the age of 65 and increases the lifetime risk by 9.5% through regulating androgen receptor pathway and the insulin-like growth factor 1 receptor pathway [2, 5]. Mutations in BRCA1 and BRCA2 have also been associated with a higher Gleason score and worsened outcomes in prostate cancer [5]. As our patient was negative for BRCA1 and BRCA2 and had a negative family history additional prostate screening was not indicated.

However, our patient was positive for minor gene mutations in the PALB2 gene and NBN gene. PALB2 disturbs BRCA2’s ability for double strand break repair (DSBR) by homologous recombination (HR) [6], while NBN gene mutations affect critical cellular functions, including the repair of damaged DNA [7]. PALB2 and NBN mutations can eventually lead to prostate cancer at a rate of 0.4% and <0.4%, respectively, but not necessarily at a rate higher than sporadic cases which is why current guidelines state that the patient should be informed of the implications of these mutations so they can make a decision regarding additional screening [8]. Compounded by the growing cost of healthcare, routine screening of minor genetic mutations is not recommended. So in unfortunate cases like our patient, who did not have major genetic mutations or a positive family history, they often fall through the screening gaps present in our current system.

## Conclusions

The relative rarity of the occurrence of PALB2 and NBN, compounded with the cost of genetic testing, results in current guidelines not recommending screening the general population for these genetic mutations. Even in the presence of minor genetic mutations, current 2018 guidelines revert to the presence of family history to guide continued screenings and prophylactic treatment. Given that our patient had no positive familial cancer history and was negative for major genetic mutations, he did not fit the mold currently in place that required early screenings. Screenings that might have caught his second primary cancer earlier. While current literature acknowledges an association between breast cancer and prostate cancer in males, further studies would be beneficial on illuminating this peculiar relationship and the growing role of rare genetic mutations and how they dictate our screening guidelines to improve patient outcomes.
